# The high price of innovation: addressing financial toxicity and healthcare inequity in gynecological malignancies

**DOI:** 10.3389/fonc.2026.1885493

**Published:** 2026-09-09

**Authors:** Montassar Ghalleb, Eya Rahmouni, Azza Chabchoub, Semia Zaraa, Yosr Zenzeri, Maher Slimane, Lamia Charfi, Lamia Naija, Tarek Ben Dhieb

**Affiliations:** 1 Surgical Oncology Department, Institut Salah Azaiez, Tunis, Tunisia; 2 Laboratory of Precision Medicine, Personalized Medicine and Oncology Investigation (LR21SP01), Tunis, Tunisia; 3 Faculté de Médecine de Tunis, Université Tunis El Manar, Tunis, Tunisia; 4 Radiation Oncology Department, Institut Salah Azaiez, Tunis, Tunisia; 5 Medical Oncology Department, Institut Salah Azaiez, Tunis, Tunisia; 6 Pathology Department, Institut Salah Azaiez, Tunis, Tunisia

**Keywords:** financial toxicities, gynecologic malignancies, radiation oncology, surgery, targeted therapy

## Introduction

Over the past decade, gynecological oncology has transitioned into an era of precision medicine, immune-targeted therapies, minimally invasive robotic surgery, and advanced radiotherapeutic modalities. While these innovations have drastically extended survival parameters, their implementation has triggered an unintended crisis: prohibitive pricing. Originally described as the material distress and psychological burden patients face during cancer care (1), “financial toxicity” (FT) is no longer a localized side effect; it is a structural obstacle to equity. Current evidence suggests that up to 50% of patients with gynecological malignancies experience significant FT, which directly correlates with compromised quality of life, sub-optimal treatment adherence, and inferior overall survival (2). As healthcare clinicians witnessing these shifting oncological paradigms, we must question the ethical dimensions of our progress. Innovation cannot be deemed clinically successful if it operates as an exclusive luxury for affluent cohorts. The international oncology community must transition away from a race for technological novelty and aggressively adopt an equitable framework that prioritizes sustainable, globally scalable patient accessibility.

## The molecular divide: the impact on pathology costs

The transition from traditional morphology-based diagnostics to molecularly driven classification has introduced a new layer of systemic FT within the pathology laboratory. Modern standards, such as the ESMO/ESGO/ESP guidelines for endometrial carcinoma, now necessitate a proactive molecular workup. This includes immunohistochemistry (IHC) for mismatch repair (MMR) proteins, p53 status, estrogen receptor status, and the costly sequencing of the *POLE* exonuclease domain (3). While these ancillary tests are essential for guiding treatment de-escalation and identifying candidates for immunotherapy, they carry significant upfront costs for equipment, specialized reagents, and bioinformatics expertise. In resource-limited settings, this creates a stark “molecular divide” where the prohibitive price of these diagnostic platforms restricts their availability to large academic centers, directly leading to the undertreatment of high-risk patients or the costly overtreatment of low-risk patients who could have safely avoided adjuvant therapy (4).

We must explicitly criticize international guideline-making bodies for establishing these highly complex diagnostic standards without concurrent, realistic economic blueprints for lower-resource health systems. When international consensus panels mandate multi-tier molecular diagnostic algorithms, they inadvertently penalize centers that cannot afford next-generation sequencing (NGS) networks, essentially codifying healthcare disparity under the guise of scientific progress. This top-down imposition of Western-centric guidelines forces clinicians in Low- and Middle-Income Countries (LMICs) into an ethical dilemma: either ignore standard-of-care recommendations or bankrupt the pathology department’s operational budget for a handful of cases.

To overcome this infrastructure bottleneck, pragmatic and scalable solutions must be aggressively integrated into regional guidelines. Utilizing Sanger sequencing to target the *POLE* exonuclease domain demonstrates that meaningful molecular classification can be achieved in resource-limited environments—a highly cost-effective approach recently validated in other LMIC settings (5, 6). Although lacking the comprehensive breadth of next-generation sequencing, Sanger sequencing remains a highly dependable alternative for detecting clinically relevant hotspot mutations, facilitating a more inclusive global genomic landscape without inducing institutional bankruptcy. It is time to champion diagnostic pragmatism over technological absolutism; Consequently, global guidelines could be designed as adaptable frameworks that support decentralized, affordable diagnostic pathways, helping to bridge the implementation gap in resource-constrained healthcare systems.

## The high price of precision: systemic therapies

The introduction of novel systemic therapeutics, such as poly (ADP-ribose) polymerase (PARP) inhibitors, antibody-drug conjugates (ADCs), and targeted immunotherapies, has radically altered the standard of care but concurrently exacerbated financial disparities. While FT in high-income nations often manifests as depleted savings (1), the consequences in LMICs are catastrophic, with objective FT prevalence reaching up to 56.96% and frequently leading to immediate household impoverishment (7). Beyond the direct costs of the drugs, hidden systemic barriers compound the financial burden. For example, immunotherapy currently lacks reimbursement coverage for metastatic endometrial cancer with microsatellite instability (MSI) in several health systems. Patients are consequently forced to navigate complex administrative processes, incur significant transportation costs, or even resort to legal action to secure treatment access following initial refusals, while completely uninsured patients face outright exclusion from accessing PARP inhibitors or immunotherapies, resulting in devastating disparities in survival and quality of life (8).

Furthermore, the pharmaceutical industry’s aggressive commercial strategy frequently shifts the cost of biological safety nets onto the public sector or the patients themselves. The hidden costs of biological monitoring required during systemic therapy—such as serum cortisol levels or troponin assays for immune checkpoint inhibitors—are often offloaded onto the patient. When public hospitals face chronic reagent shortages, patients must pay out-of-pocket at private laboratories, creating an insidious secondary layer of financial distress (9, 10).

This financial distress does not solely impact the patient; caregivers also bear an immense burden. A recent study reported that 64% of caregivers experience moderate to severe FT, resorting to depleting personal savings or selling assets to cope (11). This economic vulnerability is particularly acute among younger patients (under 65) with lower incomes, highlighting the deep socioeconomic fissures exposed by modern oncology (12). The aggressive push to market these multi-thousand-dollar monthly treatments without establishing robust, localized financial safety nets is an industry-wide structural failure. It transforms clinical breakthroughs into instruments of immediate household ruin, proving that market-driven pricing models are fundamentally unaligned with the principles of public health equity. To mitigate these stark disparities in lower-resource settings like Tunisia, multinational pharmaceutical companies have introduced specialized compassionate use and “access programs.” These corporate-sponsored initiatives provide targeted therapies at reduced costs or free of charge to selected cohorts of low-income or uninsured patients who are otherwise entirely excluded from the precision oncology market.

## Technological inequities: robotic surgery and radiation

The modernization of surgical and radiotherapeutic modalities parallels systemic therapies in generating significant economic hurdles. Robot-assisted surgery (RAS) offers undisputed clinical benefits, such as reduced morbidity and shorter hospital stays (13), yet the upfront capital required to acquire and maintain robotic systems intrinsically limits their availability. A recent nationwide cost estimation in France revealed that only 10.1% of patients undergoing para-aortic lymph node dissection for gynecologic malignancies received RAS, illustrating the restricted penetration of this technology even within well-funded High income country healthcare systems (13).

We must question whether the marginal clinical gains of robotic surgery justify the profound capital drain that starves other fundamental oncology services of necessary funding. When a single institution allocates millions to a proprietary robotic platform, it systematically depletes resources that could optimize high-volume, cost-effective standard surgical services, operating room throughput, or nursing staff retention. This resource misallocation is even more damaging when mirrored in radiation oncology.

Advanced external beam radiation and brachytherapy devices, which are essential for treating cervical cancer, remain highly centralized in elite urban hubs, this forces marginalized and rural patients to travel extensive distances, often resulting in treatment delays or reliance on suboptimal, non-standard care. In Africa, this deficit is particularly acute; recent mapping highlights a severe shortage of radiation and brachytherapy units necessary to address the continent’s growing cervical cancer burden (14). This infrastructural vacuum renders standard-of-care treatments virtually impossible for millions, embedding inequity into the physical geography of healthcare delivery.

## Bridging the gap: moving toward value-based care

Addressing these multifaceted disparities requires a critical paradigm shift: the oncology community must stop equating technological “novelty” with clinical “value.” The current trajectory of oncology innovation is financially unsustainable, particularly when newly approved, premium-priced agents often provide only marginal survival benefits (15). However, from our perspective, the implementation of value-based care must be heavily nuanced; strategies that are highly effective in HICs frequently fall short in LMICs. For instance, health economists widely advocate for tiered pricing models and managed entry agreements to reduce drug costs (16). While lowering the price of a PARP inhibitor is a crucial first step, this macro-level intervention remains fundamentally insufficient if a country lacks the baseline pathology infrastructure to test for HRD/BRCA status, or the specialized infusion centers to safely administer the drugs.

Pragmatic population-level screening and early detection strategies—such as HPV self-sampling and single-visit screen-and-treat approaches—are essential to mitigate health inequities and reduce disease burden in low-resource settings. Strengthening these programs relies on combining systematic workforce training with technological empowerment, including primary HPV DNA testing and accessible diagnostic modalities. Furthermore, integrating intelligent population management platforms enhances diagnostic accuracy, eliminates unnecessary redundant screenings, and optimizes overall cost-efficiency (17).

Therefore, true equity requires multi-tiered, context-specific interventions that address operational realities rather than theoretical market models. In High Income Countries, the focus should lean toward meso- and micro-level solutions, such as institutional decentralization of robotic surgery and routine FT screening to protect patient savings (2). Conversely, in LMICs like Tunisia, macro-level international pricing negotiations must be tightly coupled with pragmatic diagnostic workarounds and scalable screening platforms. Implementing cost-effective IHC-based molecular surrogates and utilizing localized Sanger sequencing are not merely temporary stopgap measures; they represent vital, scalable, and scientifically rigorous solutions necessary to bridge the molecular divide today. We argue that real innovation should not be measured by the complexity of an instrument, but by its capacity to deliver maximum clinical value to the widest possible patient cohort. True medical value must incorporate equity, scalability, and affordability into its fundamental definition (**Table 1**). 

**Table 1:** Multi-level framework for mitigating financial toxicity and health inequities.

Level of intervention	Proposed strategy	Objective
Policy/Macro-level	Tiered pricing models and managed entry agreements.	Align drug and device costs with clinical efficacy, cost-effectiveness, and local economic contexts.
Institutional/Meso-level	Strategic distribution and decentralization of surgical and radiotherapeutic infrastructure.	Improve geographic access to essential surgical and radiation oncology services.
Pathology, Screening & Diagnostics	Implementation of pragmatic diagnostic pathways, cost-effective IHC surrogates, localized Sanger sequencing, primary HPV DNA testing, single-visit screen-and-treat protocols, and intelligent population management platforms.	Bridge implementation gaps by adopting decentralized screening, localized molecular testing, systematic workforce training, and adaptable risk-stratification pathways, preventing redundant testing while maintaining diagnostic accuracy and population coverage.
Patient/Micro-level	Integration of routine FT screening tools and financial navigation services into clinical workflows.	Identify financially vulnerable patients early and provide proactive navigation and support.
Research/Global	Balanced alignment between technological innovation, clinical value, affordability, and population-level scalability.	Ensure research and development priorities foster a sustainable balance between innovation, clinical efficacy, affordability, and global scalability.

## Discussion and conclusion

The rapid evolution of medical and surgical treatments for gynecological malignancies is a testament to scientific ingenuity. However, high clinical success is meaningless if life-saving treatments remain out of reach for most of the world’s population. As we continue to integrate complex molecular targeted therapies, immuno-oncology agents, and advanced surgical platforms into our international guidelines, we must move beyond passive literature synthesis and confront the macro-economic and ethical architecture that perpetuates healthcare disparity.

The baseline assumption that medical innovation will naturally “trickle down” to resource-limited environments is a historical and economic fallacy. Technological innovations do not diffuse equitably; they concentrate where financial profit is maximized unless structural policies mandate their fair redistribution. By establishing centralized diagnostic criteria or endorsing high-cost therapeutics without scalable safety nets, international guideline-making bodies and regulatory organizations become passive participants in codifying healthcare disparity. True structural equity requires that health policy guidelines actively validate localized diagnostic modifications and context-specific workflows. It is insufficient to negotiate tiered drug pricing at a macro-governmental level if local institutions lack the baseline pathology infrastructure to safely identify matching cohorts, or if patients are broken by the secondary, out-of-pocket expenses of toxicological safety monitoring.

Furthermore, we must demand a profound ethical evolution in how clinical trials are designed and valued. Currently, regulatory pathways often fast-track premium-priced therapeutics that demonstrate only marginal, incremental progression-free survival benefits, completely detached from overall survival advantages or quality-of-life assessments. This commercial bias skews global research and development away from affordable, high-yield interventions. To resolve this structural crisis, global research networks, academic societies, and pharmaceutical stakeholders must actively collaborate to dismantle the economic frameworks that treat equitable cancer care as an optional commodity.

If the oncological community applies the exact same scientific rigor, structural reporting, and clinical accountability to mitigating financial toxicity as it does to managing conventional physical toxicities, systemic transformation becomes achievable. True innovation must ultimately be redefined—measured not by the complexity or premium price of a clinical tool, but by its capacity to deliver sustainable, meaningful therapeutic benefit to the widest possible global patient cohort.
